# Rapid Solidification of Invar Alloy

**DOI:** 10.3390/ma17010231

**Published:** 2023-12-31

**Authors:** Hanxin He, Zhirui Yao, Xuyang Li, Junfeng Xu

**Affiliations:** 1 School of Civil Engineering, Xi’an University of Architecture and Technology, No. 13 Yanta Road, Xi’an 710055, China; hehanxin@126.com; 2 School of Materials and Chemical Engineering, Xi’an Technological University, Xi’an 710021, China; 3 Xi’an Institute of Optics and Precision Mechanics, Xi’an 710119, China

**Keywords:** Invar alloy, solidification, undercooling, hardness

## Abstract

The Invar alloy has excellent properties, such as a low coefficient of thermal expansion, but there are few reports about the rapid solidification of this alloy. In this study, Invar alloy solidification at different undercooling (Δ*T*) was investigated via glass melt-flux techniques. The sample with the highest undercooling of Δ*T* = 231 K (recalescence height 140 K) was obtained. The thermal history curve, microstructure, hardness, grain number, and sample density of the alloy were analyzed. The results show that with the increase in solidification undercooling, the XRD peak of the sample shifted to the left, indicating that the lattice constant increased and the solid solubility increased. As the solidification of undercooling increases, the microstructure changes from large dendrites to small columnar grains and then to fine equiaxed grains. At the same time, the number of grains also increases with the increase in the undercooling. The hardness of the sample increases with increasing undercooling. If Δ*T* ≥ 181 K (128 K), the grain number and the hardness do not increase with undercooling.

## 1. Introduction

The Fe-Ni binary alloy with a content of ~36wt.% Ni is known as the Invar alloy [1]. The Invar alloy (Fe_63_Ni_36+X_) has the characteristics of a small coefficient of thermal expansion, good dimensional stability, and easy demagnetization, especially considering its very low coefficient of thermal expansion, in the range of −60~200 °C, as such material parts can maintain high dimensional stability in the range of atmospheric temperature change [2,3,4]. Since the small thermal expansion coefficient, the Invar 36 alloy mold can solve the problem of mold surface out-of-tolerance and dimensional accuracy out-of-tolerance [5]. Liu et al. studied the effect of heat input on the weld microstructure and properties in the keyhole welding of the Invar alloy using high-energy synchrotron X-ray diffraction and impact testing, finding that the grain size and texture changed significantly with the selected heat input in the fusion zone [6]. Park et al. studied the microstructure-dependent etching behavior of a partially recrystallized Invar alloy and found that surface energy, geometrically necessary dislocations, and grain boundary density were directly proportional to the etching rate [7]. Sonomura et al. studied the Invar alloy metallization of the Al_2_O_3_ substrate via friction stirring and examined the potential of the friction stirring surface treatment for the Invar alloy’s metallization [8]. Prică et al. studied the Invar alloy via mechanical alloying and obtained the alloy powders with a constant lattice parameter value up to about 350 °C [9]. Wladysiak et al. used a thermal imaging camera to analyze the solidification process of the multicomponent alloy and the effect of casting die cooling on the solidification process of the hypereutectic Al-Si alloy [10,11]. In addition, there are many other studies on Invar alloy’s application [12,13,14,15,16].

Undercooling Δ*T* (=*T_m_* − *T*_N_, where *T_m_* is the melting point of the alloy, *T*_N_ is the nucleation temperature) is the degree to which a liquid can be cooled below its melting temperature, and the highest undercooling is frequently limited by the nucleation of the solid phase around impurities or solid materials in contact with the melted material [17]. When the alloy sample starts solidifying in undercooling, due to the rapid release of the latent heat of melting, the temperature suddenly rises, and then the sample suddenly glows. The glowing process is called recalescence [18]. Although the Invar alloy has many advantages, it is easy to oxidize and difficult to achieve rapid solidification under high undercooling. Thus, there are very few reports on the rapid solidification of the Invar alloy; therefore, we still lack understanding of the rapid solidification mechanism of Invar alloys.

In this paper, the rapid solidification of Invar alloy was investigated using melt-flux technology. The thermal history curves, microstructure, and hardness of the Invar alloy under different rates of undercooling (or the recalescence degree) are shown, and then the mechanism of microstructure refinement is discussed.

## 2. Experimental Section

**Figure 1:**
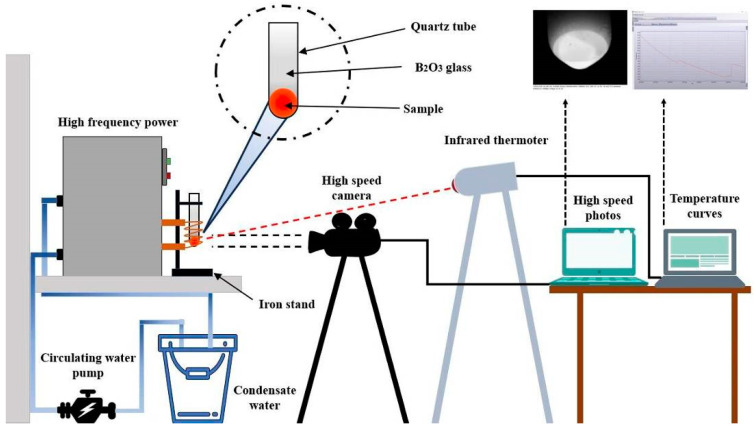
The schematic diagram of the experimental device for rapid solidification of the Invar alloy.

The Invar alloy was melted and cooled in a high-frequency induction melting furnace. In order to study the highly undercooled solidification of the Invar alloy, the schematic diagram of the experimental setup is shown in Figure 1. A sample weighing about 4 g was placed into a quartz crucible covered by small amounts of B_2_O_3_ glass, and then the crucible was placed in the induction coils of a high-frequency induction melting furnace. Then, the sample was cyclically heated and cooled to make the sample undergo solidification with different rates of undercooling. The temperature curve of each sample was monitored using a one-color pyrometer (PYROSPOT DG54N, DIAS Infrared GmbH, Dresden, Germany) with a 10 ms delay time. The solidification process was recorded using a high-speed camera (OLYMPUS I-Speed 3 MONO, Japan) with a resolution of 1280 × 1024 pixels. After solidification, the samples were sectioned and polished. And then, we etched them with a chemical solution (5 g FeCl_3_, 10 mL HCl, and 50 mL H_2_O). The microstructures were observed using optical microscopy and a scanning electron microscope. Microhardness measurements were performed at room temperature using a Vickers hardness tester, with a load of 100 g and a dwell time of 10 s.

## 3. Results

### 3.1. Cooling Curves

**Figure 2:**
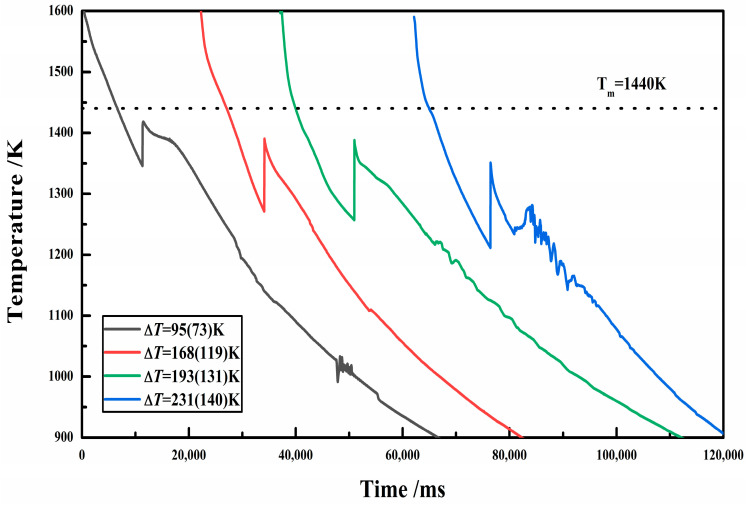
Sample cooling curves of various rates of undercooling.

The thermal history curve is important for measuring solidification undercooling. Figure 2 shows the cooling curves corresponding to various rates of undercooling. It can be seen that as the undercooling increased, the height of recalescence (*T*_R_ − *T*_N_) increased, and the maximum temperature of recalescence (*T*_R_) decreased. This indicates that the degree of solidification and non-equilibrium increased. Generally, the higher the undercooling, the faster the solidification rate, and the greater the supersaturation of the solidification structure. However, there is no exact theoretical formula for the relationship between the undercooling (=*T_m_* − *T*_N_, *T*_N_ values require temperature calibration) and recalescence height. Although the undercooling can directly reflect the non-equilibrium degree of rapid solidification, it is related to the temperature calibration method in the experiment. If the calibration method is not accurate, significant errors may occur. However, the recalescence height of solidification is a relative quantity directly measured from the same temperature curve. Whether measured from a calibrated or uncalibrated temperature curve, the change in its value can be ignored, so the influence of temperature calibration methods on the radiance can be ignored. Therefore, in order to accurately represent the phase transition temperature, we also provide the recalescence height in parentheses when displaying the undercooling values; for example, Δ*T* = 64 (42) K, where represents solification undercooling = 64 K and recalescence height = 42 K.

### 3.2. Microstructures

**Figure 3:**
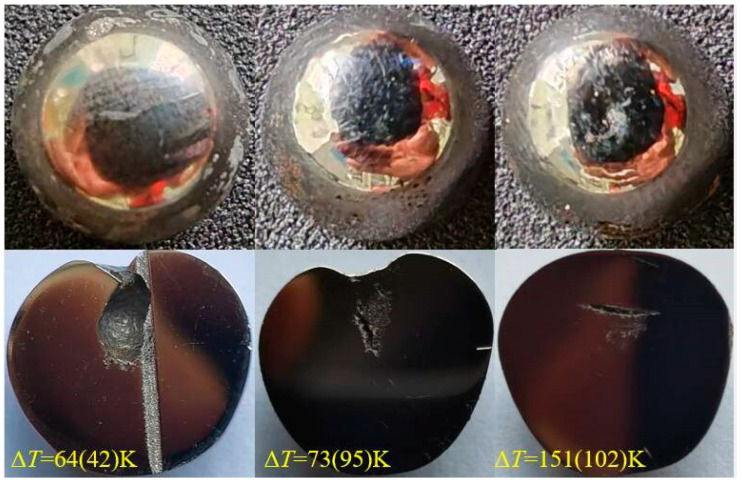
The first row shows surface images of Invar alloy samples with different rates of undercooling, and the second row shows cross-sectional images of Invar alloy samples with different undercooling degrees. It displays the shrinkage cavity at the center, which decreases with undercooling.

Figure 3 shows the surface and cross-sectional morphologies of the samples with different rates of undercooling. It can be seen that as the undercooling increases, the surface color and brightness of the sample do not change much, but the internal shrinkage pores become smaller and smaller. Generally speaking, during the alloy cooling process, the heat near the surface is dissipated quickly; thus, the sample surface solidifies first, and the interior solidifies later. Most alloy volumes shrink during solidification because the volume of the solid phase is significantly smaller than that of the liquid phase. Therefore, due to the heat dissipation sequence from the surface to the interior, the central part of the sample finally solidifies, and curing shrinkage causes the central part of the sample to shrink. In this experiment, the larger the solidification undercooling of the same sample, the smaller the central shrinkage cavity. This suggests that the larger the undercooling, the smaller the volume change is before and after solidification, or the higher the sample density, i.e., the higher the undercooling, the smaller the volume difference before and after alloy solidification.

**Figure 4:**
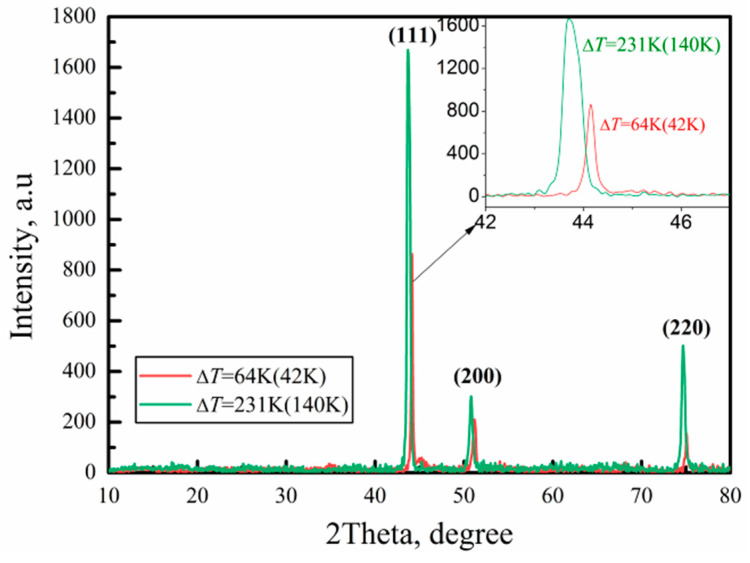
XRD analysis result of the sample with Δ*T* = 64 K (42 K) and Δ*T* = 231 K (140 K).

Figure 4 shows the X-ray diffraction (XRD) analysis of a small undercooling (Δ*T* = 64 K (42 K))solidified sample and a large undercooling (Δ*T* = 231 K (140 K)) solidified sample. It can be seen that as the undercooling increases, the position of the XRD diffraction peak shifts to the left. According to Bragg’s law, the left shift of the peak is caused by an increase in the lattice constant, which indicates the increase in solid solubility in the sample. From this result, as the solidification undercooling of the sample increases, its hardness should also increase.

### 3.3. Microstructures

**Figure 5:**
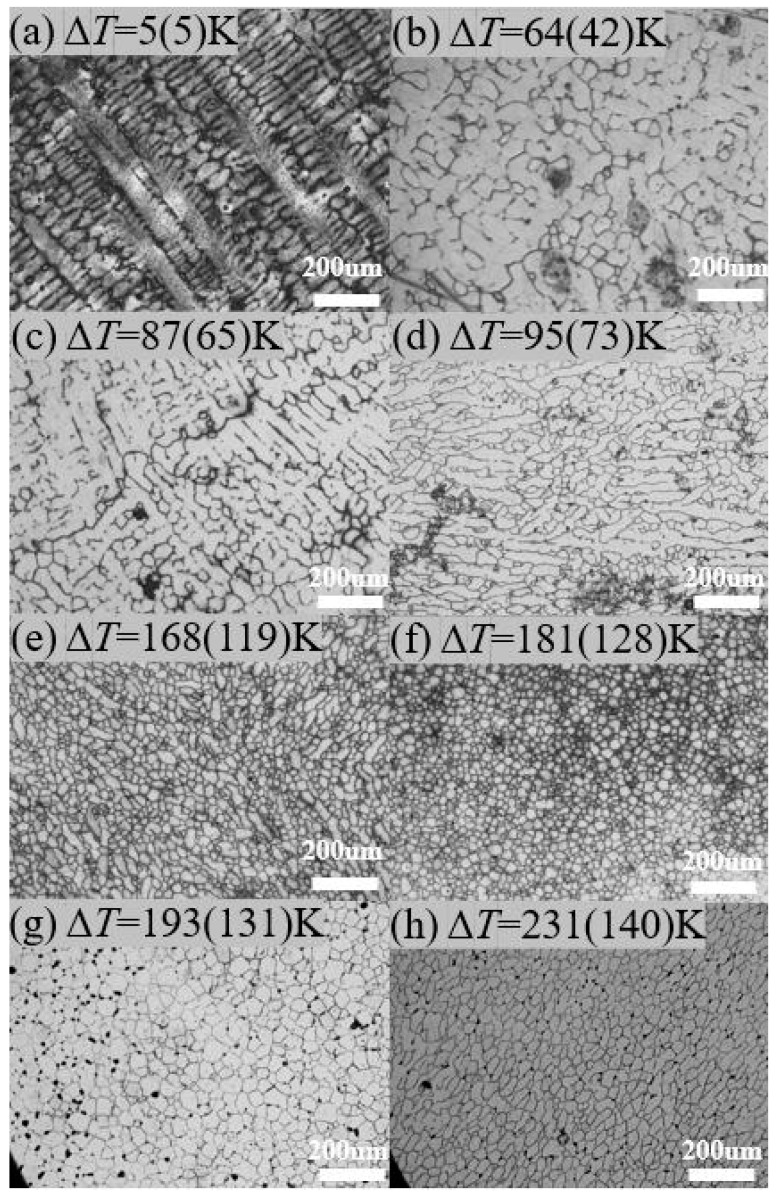
The optical microscope microstructures of the sample from different rates of undercooling: (**a**) Δ*T* = 5 K (5 K); (**b**) Δ*T* = 64 K (42 K); (**c**) Δ*T* = 87 K (65 K); (**d**) Δ*T* = 95 K (73 K); (**e**) Δ*T* = 168 K (K); (**f**) Δ*T* = 181 K (128 K); (**g**) Δ*T* = 193 K (131 K); (**h**) Δ*T* = 231 K (140 K).

**Figure 6:**
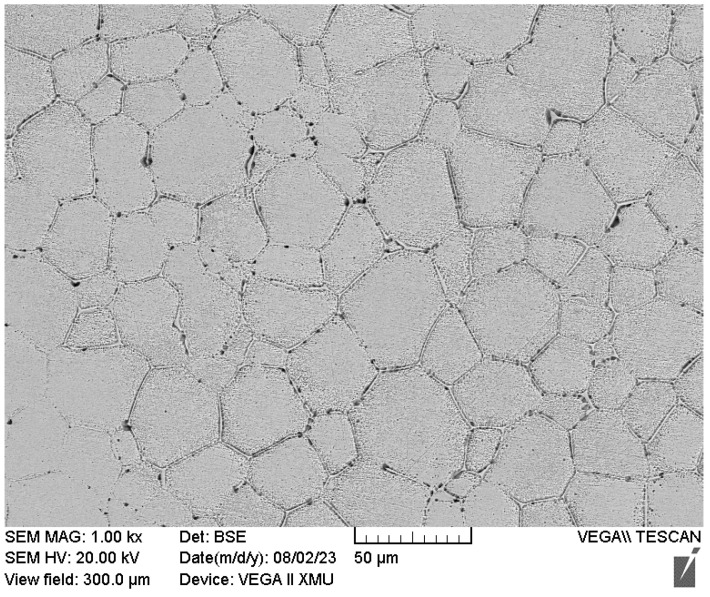
SEM image of sample with Δ*T* = 181 K (128 K).

Figure 5 shows the microstructure of samples after solidification at various rates of undercooling. As the undercooling increases, the grain size gradually becomes smaller. It is worth noting that when the undercooling increased from 181 K (128 K) to 231 K (140 K), the grain size did not continue to decrease as expected but rather increased. This may be due to the occurrence of recrystallization and growth under extremely high undercooling conditions; see the Discussion section. Figure 6 shows the SEM image of the sample with Δ*T* = 181 K (128 K), and it can be seen that the grains are equiaxed. The inconsistent grain size appears to be due to the SEM images showing specific cross-sectional areas, with some grains being cut to the top and some grains being cut to the center.

**Figure 7:**
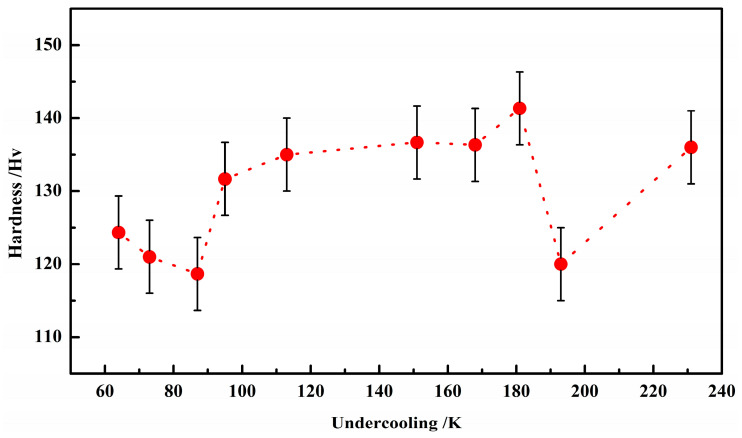
Hardness as a function of undercooling for Invar alloy.

Figure 7 shows the effect of changes in solidification undercooling on the hardness of the sample. It can be seen that as the undercooling increases, the hardness of the sample slightly increases, but if Δ*T* ≥ 181 K (128 K), the hardness of the sample suddenly decreases and then continues to increase with the increase in the undercooling. From Figure 5, Figure 6 and Figure 7, it can be observed that the finer the grains size of the sample structure, the greater the hardness of the sample. This indicates that the hardness changes in the Invar alloy conform to the Hall–Petch relationship for grain size and strength [19]. The Hall–Petch formula describes the relationship between grain size and strength, and it also holds true for hardness because there is an approximately proportional relationship between material hardness and strength. Herlach et al. reported that some alloys undergo recrystallization with solidification under high undercooling, causing the grain size to increase at critical undercooling [20]. Ma et al. also found similar phenomena to confirm that this recrystallization occurs at a higher undercooling [21].

**Figure 8:**
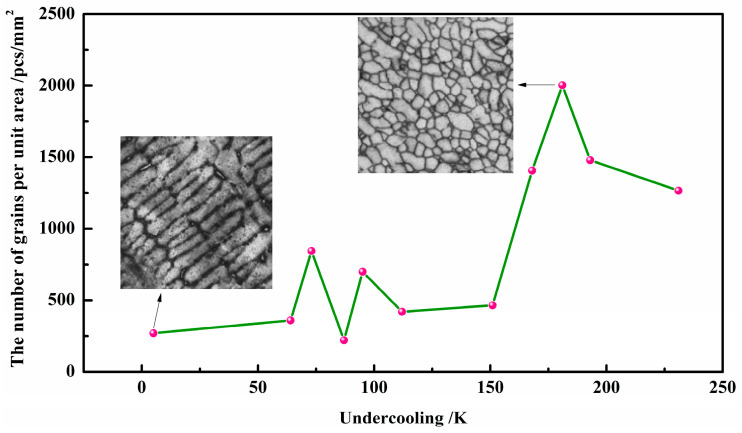
The number of grains per unit area in the microstructure as a function of different rates of undercooling.

In order to analyze the reasons for the hardness change in the sample, we further measured the number of grains per unit area of the sample, as shown in Figure 8. It can be seen that the higher the undercooling, the more grains there were. However, if Δ*T* ≥ 181 K (128 K), the number of grains suddenly decreased, which could be due to some grains undergoing recrystallization and growth. From the principle of fine grain strengthening, the finer the structure, the higher the strength and the higher the toughness of the material.

**Figure 9:**
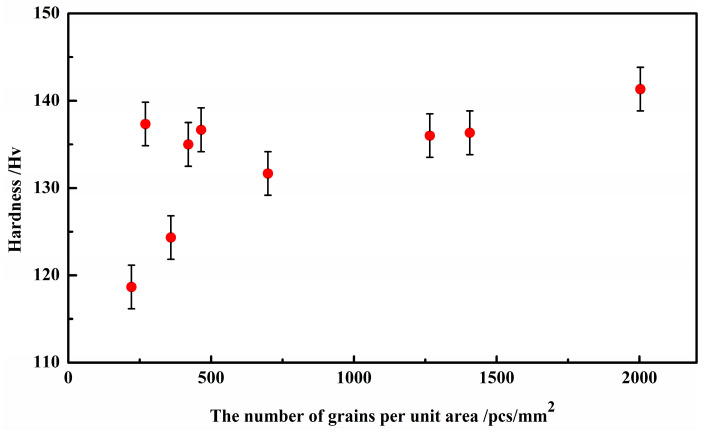
Hardness as a function of grain number per unit area.

The relationship between the grain number and hardness is shown in Figure 9. It can be seen that the more grains, the greater the hardness, indicating that hardness directly depends on the number of grains.

## 4. Discussions

The previous experimental results indicate that as the solidification and undercooling increase, the hardness of the sample also slightly increases (Figure 7), but the increased range is not large. This can be explained by the fact that as the undercooling increases, the lattice constant increases (see Figure 4), which means that the solid solubility of the crystal increases. On the other hand, from classical nucleation theory [22], the crystal nucleation rate increases with undercooling, so the number of grains increases with undercooling, leading to an increase in hardness. Therefore, the increase in the solid solubility and grain number leads to an increase in the strength and hardness of the alloy.

## 5. Conclusions

The effect of undercooling on the rapid solidification microstructure of the Invar alloy was studied using the high frequency-induced melt-flux technique. The sample with the highest undercooling of Δ*T* = 231 K (recalescence height 140 K) was obtained. It was found that the phase changed from coarse dendrites to columnar grains and to fine equiaxed grains with the increase in undercooling. The XRD diffraction peak moved to the left with increase in undercooling, indicating that the lattice constant increased. The grain number of the sample also increased with an increase in undercooling. When the sample exceeded 181 K (128 K), the grain number suddenly decreased and then increased with the increase in undercooling. In addition, the hardness also increased with the increase in the undercooling. However, the relationship between hardness and grain number is closer, and the greater the number of grains, the greater the hardness.

## Data Availability

Data are contained within the article.
